# The feasibility and acceptability of delivering and evaluating a physical activity intervention (ACCEPTANCE) for cervical cancer survivors

**DOI:** 10.1186/s40814-025-01622-8

**Published:** 2025-04-05

**Authors:** Nessa Millet, Hilary J. McDermott, Fehmidah Munir, Charlotte L. Edwardson, Tatiana Plekhanova, Esther L. Moss

**Affiliations:** 1 School of Sport, Exercise and Health Sciences, National Centre for Sport and Exercise Medicine, Loughborough University, Loughborough, UK; 2 Diabetes Research Centre, College of Life Sciences, University of Leicester, Leicester, UK; 3 NIHR Leicester Biomedical Research Centre, Leicester, UK; 4 College of Life Sciences, University of Leicester, University Road, Leicester, LE1 7RH UK

**Keywords:** Exercise oncology, Feasibility trial, Process evaluation, Cervical cancer survivorship

## Abstract

**Background:**

Cervical cancer treatment can be associated with long-term physical, psychological and emotional issues leading to compromised quality of life (QOL). Physical activity (PA) may improve QOL in cervical cancer survivors.

**Methods:**

A one-arm pre/post-feasibility study with parallel process evaluation was conducted to test the feasibility and acceptability of a 12-week PA intervention (ACCEPTANCE). Feasibility was determined by using pre-established criteria relating to inclusion criteria, recruitment and retention, compliance with intervention components and compliance with evaluation measures. The 12-week intervention consisted of (1) an online education session on the benefits of PA and a barrier identification and problem-solving session; (2) a *Fitbit* monitor; (3) daily and weekly diary entries; (4) brief fortnightly online health coaching sessions; and (5) organising and participating in group walks; (6) online social interaction via a messaging group. Measures included accelerometer assessed PA and sleep, quality-of-life and PA behaviour change questionnaires distributed at 4-time points (baseline, week 6, week 12, and week 24). Process evaluation measures were questionnaires on the acceptance of intervention components and semi-structured interviews with participants after completion.

**Results:**

Thirty participants were recruited with a retention rate of 77% at week 24. The education session, health coaching and *Fitbit* PA monitor were deemed to be feasible based on compliance rates whilst the online messaging group, diary and group walking compliance suggested that modifications would be needed for these components. Accelerometer and questionnaires were deemed to be feasible evaluation measures based on compliance rates. Process evaluation results suggest that the *Fitbit* and health coaching sessions were perceived to be the most beneficial aspects of the programme as they enabled self-monitoring of behaviour and goal setting. Unexpected mechanisms of behaviour change were the integration of a small change approach and formation of habits to facilitate PA, whilst barriers to behaviour change included the online, virtual nature of the programme, poor mental health, and environmental challenges. PA levels were higher after the intervention, whilst global QOL was seen to worsen throughout the study, and depression and anxiety symptoms improved.

**Conclusions:**

The ACCEPTANCE study is deemed to be feasible based on criteria established a priori, and only minor modifications would needed to implement in a definitive randomised control trial. The intervention showed potential to increase PA, through hypothesised mechanisms of self-monitoring behaviour and problem solving and unexpected mechanisms such as habit formation.

**Trial registration:**

ISRCTN16349793, Registered 30 September 2020.

**Supplementary Information:**

The online version contains supplementary material available at 10.1186/s40814-025-01622-8.

## Key messages regarding feasibility

- This study was designed to test the feasibility of delivering and evaluating a 12-week physical activity intervention in women treated for cervical cancer.
- Feasibility was assessed using pre-determined criteria. Recruitment and retention rates were deemed to be feasible. The inclusion criterion which excludes those meeting the current national physical activity guidelines requires modification to optimise greater representation of women treated for cervical cancer in a subsequent trial. The intervention launch and education session, the *Fitbit* monitor and health coaching were deemed feasible, whilst the group walking, the messaging group and the diary require modification prior to future implementation. A research-grade accelerometer and questionnaire booklet administered at four time points throughout the study were deemed to be feasible.
- Only minor modifications in the study methods have been indicated before ACCEPTANCE can be adapted to a definitive randomised control trial.

## Introduction

Although cervical cancer (CC) incidence is falling in many countries [1], for those individuals who are diagnosed it can have a life-changing and potentially life-long impact [2]. Post-treatment morbidity can include fatigue, bladder/bowel dysfunction [3], neuropathy [4], sexual dysfunction [5], lymphedema and pelvic pain. Psychological and cognitive impacts are also frequently reported following a CC diagnosis [6, 7], which is not confined to cases of advanced disease, but also affects patients diagnosed at an early stage [8]. As a result, quality of life (QOL) can be negatively impacted [6, 9] and patients who have undergone treatment for CC are documented to have a lower QOL compared to healthy controls [10, 11] and patients treated for other gynaecological malignancies [12, 13].

The age of patients impacted by CC is typically younger than those diagnosed with ovarian, uterine or vulval malignancy, with a peak age incidence in the UK of 30–34 years [14]. Previous work indicates that the aetiological association of CC with the Human Papillomavirus (HPV) can result in feelings of blame, guilt and stigma which may impact psychosocial recovery [8], whilst patients who undergo CC present with more unmet psychological and emotional needs compared to those treated for breast cancer [15]. In addition, CC survivors have reported how a lack of stage-matched peer support can contribute to the creation of unrealistic expectations of the recovery journey [8].

CC survivors are underrepresented in interventions to improve post-treatment QOL [16, 17]. A development study which followed the intervention mapping approach [4] identified physical activity (PA) participation as a viable mechanism to improve QOL after CC. There is an abundance of literature supporting PA interventions after cancer. Reported benefits include reduced fatigue [18, 19], weight loss [20], increased aerobic capacity and cardiovascular health, improved psychological outcomes [21], including symptoms of depression [22], and anxiety [23], along with social benefits [24]. Despite this, levels of PA after gynaecological cancers are low [25–27] whilst data on the proportion of CC survivors achieving national PA guidelines has not been reported. As a result, little is known about the types, duration or frequencies of PA that are feasible and acceptable following CC treatment. 

Therefore, this study was designed to explore the feasibility and acceptability of delivering and evaluating a multi-component behaviour change intervention focused on increasing PA after CC. The intervention was named ACCEPTANCE (Acceptability in Cervical Cancer of an Exercise Programme Tailored to An oNline Community Environment) and its development was informed by models and relevant theories of behaviour change [4].

## Methods

### Patient and public involvement

Patient and public involvement (PPI) has informed the development of the research since its inception. Details on the involvement and modes of PPI integration are detailed in the study protocol [28].

### Study design

A single-arm pre-/post-feasibility study with parallel process evaluation was conducted through the University Hospitals of Leicester (UHL) NHS Trust, in collaboration with Loughborough University and the University of Leicester in the UK. Ethical approval was granted by the West of Scotland Research Ethics Committee 1 (20/WS/0062) in July 2020. This feasibility trial is reported in line with CONSORT guidelines extended for feasibility trials [29] (Supplementary file S1). The trial registration number was ISRCTN16349793, registered on 30 September 2020.

### Sample size justification

The recruitment target was 30 participants, after which recruitment efforts were stopped. The sample size of 30 participants was chosen primarily as it was a realistic target when considering cervical cancer incidence both in the UK and at UHL where recruitment took place. It was also determined based on practical considerations of financial, time and labour resource capacities. Previous feasibility studies of lifestyle and physical activity interventions in endometrial and breast cancer have employed similar sample sizes [30, 31]. Additionally, a sample size of 30 participants affords an adequate precision around the pre-specified retention rate of 70% when applying a 17% margin of error with 95% confidence [32, 33].

### Participants

Eligible participants were those aged between 18 and 60 years who had undergone treatment for CC at least 6 months prior or more with curative intent (either surgery, chemoradiotherapy or both modalities) and who were not meeting the national PA guidelines, defined as 150–300 min of moderate to vigorous intensity PA (MVPA) per week [34]. PA-level eligibility was assessed using the Scottish Physical Activity Questionnaire [35]. The age range of 18–60 years was chosen to reflect the Chief Medical Officer (CMO)’s PA adult guidelines which are recommended for adults aged 18–65 years, and to optimise engagement and adherence to a PA programme delivered online [36, 37]. Exclusion criteria were clinical/radiological evidence of disseminated malignancy; pregnancy or breastfeeding; WHO performance status ≥ 3; comorbidity that in the opinion of the patient’s supervising gynaecologist/oncologist would preclude the patient from meeting the study PA requirements; and not resident within the Midlands region in the UK.

### Recruitment

Participant recruitment took place via two streams, either (1) patients under clinical follow-up, or (2) advertisements (physical and online) in the general public domain. Those interested were provided with details of the study and screened for eligibility. Consent procedures took place virtually after which all participants were asked to return a signed consent form by post.

### Intervention

Details on the intervention development [4] and the study protocol including feasibility objectives [28] have been published previously. Considering the diminished QOL after cervical cancer treatment, the logic model of the problem (i.e. low PA participation in the target group) was developed (Supplementary file 2.4). In brief, the aim of the multi-component intervention was to increase PA levels of the target population, specifically through individual and group-based walking over 12 consecutive weeks. The programme components are underpinned by social cognitive theory (SCT) [38] and informed by the health belief model [36] and theories of self-regulation [39] which were determined via the development of a logic model of change (Supplementary file S2.5).

Participants were firstly allocated into intervention groups of 3–6 people each based on their geographical location. The intervention included group online education provision, problem-solving, barrier identification, and goal setting in relation to increasing PA levels after treatment for CC. Intervention launch sessions were intended to be delivered in person; however, due to restrictions posed as a result of COVID-19, this was adapted to an online session delivered to each participant group. Self-monitoring of PA behaviour was facilitated by providing participants with a programme diary to complete daily and weekly, and a consumer PA device (*Fitbit inspire*). Throughout the study, peer support through social interaction and group walking amongst participants was encouraged via a messaging platform, which allowed participants to maintain contact and organise group walking sessions. NM prompted group walk organisation via the messaging platform. Participants were instructed and encouraged to post their PA achievements on the online group and to update others in the intervention group on their progress, thereby creating opportunities for social interaction. Individual goals were reviewed fortnightly through online health coaching sessions based on the GROW model [40]. These sessions took place online via video/telephone call administered by the researcher (NM).

### Data collection

Participant recruitment, eligibility, and retention rates were collected. Measure completeness (measures completed and returned to the research team) and compliance rates (valid data) were monitored at baseline, week 6, week 12, and week 24. Where possible, reasons for withdrawal were also recorded. Demographic data, treatment-related information and medical history were collected at baseline. Data were collected to describe the launch and education attendance rates, the number of diary entries completed, the number of health coaching sessions attended, the number of participants who posted on the online forum and engaged with the group messaging, the number of participants who took part in a group walk and the frequency of these.

A research-grade blinded accelerometer (GENEActiv Original, Activinsights Ltd., Cambridgeshire, UK) was worn by participants on their non-dominant wrist continuously for 8 days at four evaluation time points (baseline, week 6, week 12, and week 24) to measure PA and sleep. Accelerometers were configured to record at a frequency of 100 HZ. Data from the accelerometer were downloaded using GENEActiv PC software V.3.2 and accelerometer files were processed with R-package GGIR version 2.4–0 [41]. Files were autocalibrated and the magnitude of dynamic acceleration (Euclidean norm minus one in mg, ENMO) averaged over 5-s epochs. Non-wear was imputed using the default setting, that is, invalid data were imputed by the average at similar time-points on different days of the week. Participants were excluded if post-calibration error was > 0.01 g (10 mg), they had < 3 days of valid wear (defined as ≥ 16 h per day), or if wear data were not present for each 15-min period of the 24-h cycle. Sleep logs were used to guide the algorithm to identify the sleep window. Variables of interest were Volume of PA (average acceleration; PA across the whole day measured in mil-li-gravitational units; [mg]), MVPA (the time accumulated in 1-min bouts of > 100 mg), Light PA (the time accumulated with acceleration between 40 and 100 mg), Sedentary behaviour (the time accumulated during the waking day below 40 mg), Sleep duration and Sleep efficiency (the ratio of sleep duration to the duration of the sleep window).

A questionnaire booklet was also administered at baseline, week 12, and week 24. The booklet contained the self-efficacy for walking scale (SEW) [42], PA Enjoyment Scale (PACES) [43], European Organisation for Research and Treatment of Cancer core Quality of Life questionnaire (EORTC QLQ-30), Menopausal Rating Scale [44], Hospital Anxiety and Depression scale (HADS) [45] and the Fatigue Symptom Inventory (FSI) [46]. These constructs were measured as they were identified within the logic model of change and thus would be hypothesised to change in a definitive trial.

### Process evaluation

A mixed methods process evaluation was conducted following Medical Research Council (MRC) guidance [47], to understand how the ACCEPTANCE intervention was delivered and its acceptability amongst CC survivors. Questionnaires were developed to evaluate various aspects of the programme including the intervention launch and education session, acceptance of wearing and engaging with the *Fitbit* activity monitor (administered at week 12 and week 24), and experiences of using the online group messaging platform. A researcher log was completed by NM to record reflections of interactions with participants throughout the duration of the study. Feedback was divided into four categories at the time of collection, including acceptance of programme components; acceptance of intervention evaluation; alterations to future delivery, and barriers to PA. A semi-structured qualitative interview (telephone or virtual video) was conducted with participants after the 6-month evaluation measures to investigate the feasibility and acceptability of the programme components. The evaluation interview schedule (available in Supplementary material S2.1) asked participants about the following: perceptions of the intervention; benefits of participation; experiences of the group walking; reasons for withdrawal (where relevant), maintenance of intervention components and PA behaviour.

### Data analysis

Quantitative data was analysed using IBM SPSS statistics 25. Feasibility thresholds were determined a priori in the study protocol using relevant guidance [28, 48]. Means and standard deviations (SDs) were used to describe PA and sleep metrics from the accelerometer at each evaluation time point (baseline = T0; week 6 = T1; week 12 = T2; week 24 = T3). Constructs measured via questionnaire were computed using standard scoring procedures. Change scores were calculated for each outcome for participants who provided data at each time point: T0 and T1; T1 and T2; T2 and T3; T0 and T3. Due to data skewness, only responses from the process evaluation questionnaires representing either “strongly agree” or “agree” for each outcome are reported.

Qualitative data were analysed using template analysis [49], a form of thematic analysis which incorporates a large degree of structure in the analysis, due to the development of a priori themes to guide the analysis process. A priori themes or categories were developed pragmatically using the MRC guidance to reflect the distinct needs of the research and the type of data collected:

1. *Delivery*: establishing whether the intervention was delivered per protocol, any deviations, and the reasons for and outcomes of these deviations.
2. *Views on the intervention*: participant experience of the intervention.
3. *Mechanisms of impact*: exploring hypothesised and unintended impacts of the programme.
4. *Contextual factors****:*** understanding the context in which the intervention was delivered.

Interpretations of qualitative data and the study findings more broadly were discussed with PPI members and within the research team to enable the generation of conclusions which transparently and credibly reflect the experiences of participants. Full details of the qualitative and quantitative process evaluation methods are published separately [28] and are available in the associated PhD thesis [50].

## Results

### Feasibility and acceptability of the intervention

#### Study eligibility, recruitment, and retention

**Fig. 1:**
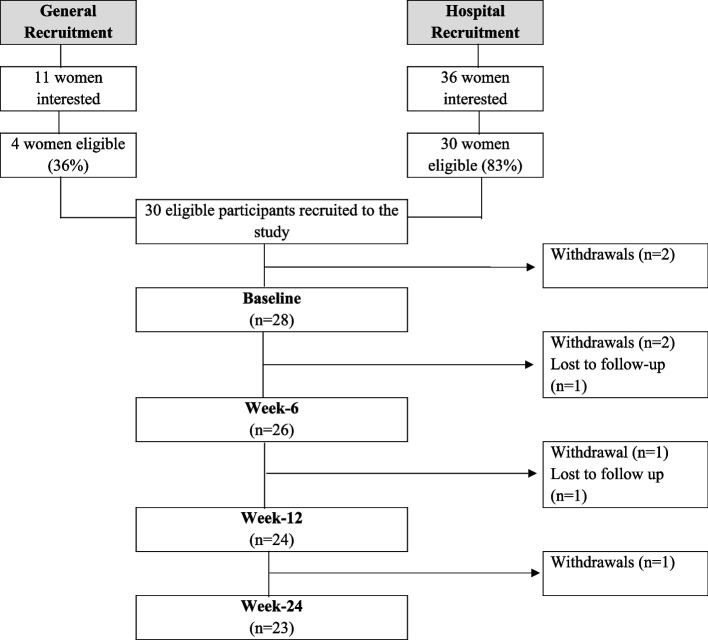
Flowchart representing participant numbers and measure completeness at each time point

Forty-seven women expressed an interest in participating in the study, 36 from the hospital recruitment stream (76.6%) and 11 from general recruitment. Of these, 34 individuals were eligible (64%), with the reasons for ineligibly including: already achieving PA guidelines (*n* = 6); less than 6 months post-treatment (*n* = 1); no cervical cancer diagnosis (*n* = 2); and living outside of the study geographical location (*n* = 4). In total, 30 participants were recruited and consented between October 2020 and April 2021. Reasons for non-participation of the 4 eligible women could not be obtained and a further 2 participants withdrew prior to baseline assessment. Participant demographic information provided at baseline is available in Table 1. The retention rate at week 24 was 76.7%, with 5 participants withdrawing from the study and 2 lost to follow-up (Fig. 1). Reasons for withdrawal were: injury unrelated to the programme (*n* = 3), a lack of time (*n* = 1), and a medical issue (*n* = 1). A participant flow diagram is displayed in Fig. 1 showing attrition throughout the study.

**Table 1:** Baseline demographic characteristics

Demographics		*N* (% of total sample)	M (SD)
Age (years)			40 (7.95)
Ethnicity	White British White Other Black African	26 (92.9%) 1 (3.6%) 1 (3.6%)	
Employment status	Unemployed Part-time Full-time (fixed shifts)	5 (17.9%) 5 (17.8%) 18 (64.3%)	
Marital status	Married Single	19 (67.9%) 9 (32.1%)	
Treatment modality	Local excision Trachelectomy Hysterectomy Surgery + radiotherapy Surgery + chemoradiotherapy Chemoradiotherapy	6 (21.4%) 3 (10.7%) 11 (39.3%) 1 (3.6%) 2 (7.1%) 4 (14.3%)	
Time since treatment	< 1 year 1–2 years 2–5 years 5–10 years	2 (7.1%) 16 (57.1%) 1 (3.6%) 9 (32.1%)	
Lymphadenectomy	Yes	12 (42.9%)	
History of depression	Yes	8 (28.6%)	

*Key*: *M *mean, *SD *standard deviation

### Evaluation measures

Compliance and completeness rates with evaluation measures are displayed in Supplementary file S2.2. Compliance (> 3 days of data) with wearing the accelerometer was very high, although it fell from 100% at baseline to 78.3% at week 24. Completeness of the questionnaire booklets was also high with the lowest completeness rate seen on the MRS at week 6. How results relate to feasibility criteria established a priori are detailed in Table 2.

**Table 2:** Feasibility outcomes, percentage thresholds, feasibility results and implications for a definitive trial

Outcome	Criteria for determining feasibility	Result	Implications for a definitive trial
Feasibility of inclusion criteria	If an eligibility criterion is responsible for more than 40% of non-eligibility, the eligibility criterion/criteria will be reviewed prior to future implementation	Meeting PA guidelines = 50% non-eligibility Located outside of East Midlands = 26.3% non-eligibility Non-cancer diagnosis = 16.6% non-eligibility Treated less than 6 months prior to recruitment = 8.3% non-eligibility	Feasible with modifications Feasible as is Feasible as is Feasible as is
Feasibility of study recruitment	A recruitment success rate is either: The recruitment of 30 participants in the first 3 months OR 75% of those identified as eligible are recruited	26 participants were recruited in the first 3 months 88% of those eligible recruited in the first 3 months	Feasible with close monitoring Feasible as is
Feasibility of study retention	A successful retention rate at week 24 was 70%	77% of participants retained at week 24	Feasible as is
The feasibility of and compliance with intervention components	The threshold of feasibility for these rates will be 70% of participants complying respectively	93% compliance with programme launch 88% attended at least 70% of health coaching sessions 95% compliance with *Fitbit* PA monitor at week 12 64% posted on online messaging group 58% compliance with diary 43% compliance with group walk organisation and attendance	Feasible as is Feasible as is Feasible as is Feasible with modifications Feasible with modifications Feasible with modifications
The feasibility of the evaluation measures	Compliance with evaluation measures will be deemed successful if no more than 20% of participants fail to provide questionnaire and accelerometer data at both baseline and week 24 follow-up	Accelerometer: 100% baseline compliance; 91.3% week 24 compliance Questionnaire measures: 92.8% baseline compliance; 86.9% week 24 compliance	Feasible as is Feasible as is

Process evaluation data indicated that the study measures (accelerometers and questionnaires) were generally well accepted; however, some participants reported that some of the questions impacted them negatively, for example:

Participant struggled to complete the questionnaires, relating this to her poor mental health. She found the questions difficult to understand and found that answering the questionnaire negatively impacted her mood and anxiety. She felt that when she did attempt to answer the questionnaire her answers were not accurate as she would try to protect herself by answering incorrectly (Researcher log, week 13)

This insight also indicates that the validity of certain measures may be compromised within participant groups who report pre-existing mental health problems or where the questions asked reinforce negative feelings.

### Intervention components

Adaptations.

Protocol adaptations are any intentional changes or alterations made to the protocol during its delivery to enhance contextual fit [46]. Only one protocol adaptation arose during the implementation of the ACCEPTANCE programme. It became apparent that participants did not have a means of measuring their levels of MVPA. This was despite the aim of the programme being to gradually increase PA levels in line with the national recommendations (150–300 min of MVPA per week). Therefore, it was decided that during the week 6 health coaching session, device-assessed MVPA would be fed back to participants to increase their knowledge of how their activity levels mid-intervention compared with the national recommendations.

Acceptability.

All 28 participants attended the virtual intervention launch and education session, whilst 25 (88%) attended at least 70% of health coaching sessions, and 14 (58%) reported completed diary entries for all 12 weeks. Twenty-three (82%) participants created a profile on the online messaging platform and of these, 18 (64%) posted within their respective group. The mean number of posts made by a participant over the 12 weeks was 9 (SD = 17.48). Four group walks took place over the 12 weeks including 2–3 participants per walk, with a total of 10 (43%) participating in at least one walk. The implications of these results on markers of feasibility are detailed in Table 2.

Mixed methods process evaluation data described the intervention as motivating, facilitative and challenging, exampled by the following participant who took part despite experiencing lower leg neuropathy:

Absolutely loved it. It was the most motivated I'd been in ages because people before have been so quick to sort of say…“Just do what you can, if you if you get out of bed in the morning, then that's all you need to do”. That's not enough for me, so it was so nice to have someone going…. “Yeah, I mean that was good, but can you do like five-thousand more next week?” (P8)

Walking was a well-liked form of PA and those who partook in a group walk reported enjoying the experience and found that socialising with peers was highly beneficial: “it's just really nice to be able to talk to someone who had been in the same situation” (P6). However, a lack of perceived competence and self-esteem were barriers to group walk participation and it was suggested that group walking could be improved if groups were treatment-matched. “I mean they both have the operations, whereas I hadn’t, so perhaps you know they're not up to doing as much as what I was doing” (P15), and ‘Participant reflected that no other women in her group had the same treatment type as her which limited their understanding of her experience. She has suggested that separate stage matched messaging groups be created’ (Researcher log entry, week 8).

Participant feedback collected via process evaluation questionnaires is detailed in Table 3. There was concordant agreement between quantitative and qualitative data to suggest that the *Fitbit* was the most enjoyed aspect of the programme as it provided participants with prompts to be active and increased knowledge of their steps per day which enabled goal setting: “The Fitbit has been amazing. I feel like I've carried on like monitoring stuff personally …all of the things that I’ve found out” (P11). Despite the intervention launch and education session being perceived as beneficial in terms of information provision, participants reflected on a missed opportunity for social interaction due to the virtual nature of the session: “It might have been better if we could've met in person because I think you can have different interactions and you get to know a bit of their personality as well, rather than just seeing them on a screen” (P5).

**Table 3:** Process evaluation questionnaire results

Statement			
	Agree *N* (%)	Strongly agree *N* (%)	Total agree *N* (%)
The intervention launch and education session…(N=20)			
was enjoyable	7 (35%)	13 (65%)	20 (100%)
length was appropriate	4 (20%)	16 (80%)	20 (100%)
level was appropriate	7 (35%)	13 (65%)	20 (100%)
was motivational	6 (30%)	14 (70%)	20 (100%)
provided adequate information about the benefits of PA	7 (35%)	13 (65%)	20 (100%)
Encouraged me to set PA goals	8 (40%)	12 (60%)	20 (100%)
Increased my awareness of barriers to PA	9 (45%)	11 (55%)	20 (100%)
The* Fitbit* monitor…(N=18)			
was easy to use	6 (33%)	11 (61%)	17 (94%)
was convenient when carrying out daily activities	5 (27.7%)	7 (39%)	12 (66.7%)
has made me more aware of how much physical activity I do	5 (27.7%)	11 (61%)	16 (88.9%)
has encouraged me to increase my physical activity	7 (39%)	9 (50%)	16 (88.9%)
Provided prompts which encouraged me to be physically active	8 (44%)	6 (33%)	14 (77.8%)
The messaging group…(N=18)			
was an effective means of organising group walks	6 (33%)	2 (11%)	8 (44.4%)
	No, *N* (%)	Yes, *N* (%)	
I felt comfortable posting on the messaging group	6 (33%)	12 (67%)	
Were you involved in the organisation of any group walks?	8 (44%)	10 (56%)	

Health coaching was well accepted, with participants reporting that they found the fortnightly sessions motivating and an important opportunity for self-reflection. It was also suggested that these sessions acted as a form of social support during COVID-19 restrictions in the absence of group walking with other participants: “Covid fought hard against the group support, but in terms of support network from me to you, that was invaluable, and I found that reallybeneficial” (P16). On the other hand, 10 out of 18 participants disagreed that the messaging group was an effective means of organising group walks (Table 3), exampled qualitatively: “You know nobody really bothered, and I found that a bit of a waste of time”. It was suggested that organisation of walks would have been more effective had they been researcher-led rather than peer-led:

I think for me if somebody had sort of taken the lead or you know if you had said…right, on this date why don't we all meet up? Because it felt like every time we tried to organize a meet up it was like…well I can't really do this, then I can't really do that (P8).

This is further supported by one-third of participants reporting that they did not feel comfortable posting on the online messaging platform (Table 2), which was a barrier to virtual communication and peer support.

Finally, the diary was viewed as a useful reflection tool, for example, “I did briefly reflect on the diary and went back on it from different weeks and I think my mood got better throughout the course” (P21), although weekly entries to rate psychological/physical symptoms were perceived as challenging due to fluctuations throughout the week.

## Potential changes to behaviour and health

### PA, sleep, and wellbeing measures

The volume of PA, MVPA and light activity increased overall from baseline to week 24, whilst sedentary time was lower at week 24 compared to baseline. There were very small changes in sleep duration and sleep efficiency scores which were seen to decrease from baseline to week 24 (Table 4).

**Table 4:** PA outcomes measured at 4 time points and associated change

	T0 (*n* = 28)	T1 (*n* = 24)	T2 (*n* = 19)	T3 (*n* = 18)	*T1–T2* (*n* = 24)	*T2–T1* (*n* = 17)	*T3–T2* (*n* = 18)	*T3–T0* (*n* = 18)
	M (SD)	M (SD)	M (SD)	M (SD)	M (SD)	M (SD)	M (SD)	***M*** (SD)
*Device assessed PA*								
Volume of PA (m*g*)	24.58 (8.23)	25.94 (8.00)	28.66 (9.58)	28.40 (7.37)	1.36 (3.52)	2.71 (5.52)	− 0.25 (5.18)	3.82 (5.78)
MVPA (min/day)	25.65 (31.74)	29.87 (25.31)	39.72 (8.07)	37.11 (22.67)	4.22 (20.61)	9.84 (19.24)	− 2.61 (16.14)	11.45 (24.94)
Light activity (min/day)	225.71 (71.87)	240.44 (69.98)	258.21 (79.24)	252.95 (71.03)	14.73 (56.02)	17.77 (49.21)	− 6.26 (36.47)	26.24 (45.49)
Sedentary time (min/day)	657.60 (101.55)	615.03 (99.57)	625.66 (113.71)	634.12 (90.35)	− 42.57 (67.85)	10.63 (101.22)	8.47 (83.30)	− 23.47 (97.17)
*Device assessed sleep*								
Sleep duration (h/day)	6.76 (1.03)	7.10 (1.05)	6.07 (1.36)	6.31 (1.11)	0.34 (0.84)	− 0.10 (1.05)	0.24 (1.14)	− 1.47 (1.22)
Sleep efficiency (%)	86.93 (6.60)	86.55 (7.54)	81.49 (9.08)	86.26 (6.99)	− 0.39 (5.41)	− 5.06 (7.23)	4.77 (7.36)	− 1.18 (8.55)

Key: *T0 *baseline, *T2 *week 12, *T3 *week 24, *M *mean, *SD *standard deviation, *n *number of participants who provided data at a given time point. T2–T0, T3–T2, T3–T0 represent change scores; change variables refer to participants who provided data at both time points. *Volume of PA (average acceleration)*: PA across the whole day measured in milli-gravitational units (mg); *MVPA*=moderate to vigorous PA, the time accumulated in 1-min bouts of > 100 mg; *Light PA:* the time accumulated with an acceleration between 40 and 100 mg, *Sedentary behaviour* The time accumulated during the waking day below 40 mg; *Sleep efficiency*: the ratio of sleep duration to the duration of the sleep window

### Well-being

Scores on the EORTC global QOL subscale decreased from baseline to week 24. The following EORTCQLQ-30 subscales worsened from baseline to week 24: emotional functioning, physical functioning, cognitive functioning, social functioning, role functioning, and fatigue. In contrast, improvements from baseline to week 24 were seen with: pain and insomnia symptoms. Global QOL and insomnia symptoms represent the largest changes seen from baseline to follow-up (Table 5). Menopause-related QOL decreased from baseline to week 24 (Table 6). Both the depression and anxiety subscales of the HADS questionnaire saw overall improvements in symptomology scores from baseline to week 24, whilst fatigue scores also improved from baseline to week 24.

**Table 5:** Clinical outcomes measured at 3 time points and associated change scores

	T0 (*n* = 28)	T2 (*n* = 19)	T3 (*n* = 18)	T2–T0 (*n* = 19)	T3–T2 (*n* = 19)	T3–T0 (*n* = 19)
	M (SD)	M (SD)	M (SD)	M (SD)	M (SD)	M (SD)
*EORTCQLQ-30*						
Quality of life	66.00 (18.47)	64.04 (22.58)	57.89 (22.13)	− 2.19 (28.71)	− 6.14 (23.71)	− 8.33 (33.32)
Emotional functioning	70.67 (22.32)	74.56 (27.98)	69.30 (30.44)	3.95 (26.70)	− 5.26 (18.68)	− 1.32 (23.78)
Physical functioning	93.07(10.27)	90.88 (10.93)	91.58 (10.15)	− 3.86 (10.50)	0.70 (8.86)	− 3.16 (8.10)
Cognitive functioning	74.00 (25.50)	82.46 (23.88)	70.18 (40.28)	8.77 (23.81)	− 12.28 (39.61)	− 3.50 (45.67)
Social functioning	78.00 (30.32)	87.72 (19.12)	77.19 (24.92)	5.26 (28.36)	− 10.53 (30.54)	− 1.75 (30.46)
Role functioning	83.33 (22.57)	86.84 (24.58)	84.21 (25.14)	0.88 (26.34)	− 2.63 (29.53)	− 1.75 (29.34)
Fatigue symptom	28.44 (21.30)	27.49 (26.29)	32.26 (25.63)	− 1.17 (26.34)	3.51 (29.54)	4.68 (29.34)
Pain symptom	28.00 (26.67)	28.95 (22.80)	25.44 (28.53)	6.14 (15.92)	− 3.51 (19.70)	− 2.63 (21.70)
Insomnia symptom	46.67 (28.87)	31.58 (26.00)	38.60 (29.94)	− 17.54 (28.04)	7.08 (19.70)	− 7.01 (21.7)
*HADS*						
Depression	8.96 (1.79)	7.95 (1.78)	8.16 (1.77)	− 1.05 (2.86)	0.21 (1.75)	− 0.84 (2.83)
Anxiety	10.32 (4.19)	8.05 (5.17)	7.68 (4.68)	− 2.42 (3.97)	− 0.37 (2.19)	− 2.79 (3.22)
* Fatigue*	20.04 (13.62)	12.32 (14.42)	15.42 (14.84)	− 7.72 (18.13)	3.1 (14.94)	− 4.62 (9.77)
* PA enjoyment*	69.65 (19.40)	76.44 (22.15)	76.28 (23.60)	5.47 (19.18)	− 0.17 (16.76)	5.23 (21.76)
* Self-efficacy for walking*	59.20 (29.33)	66.22 (25.57)	67.74 (28.73)	6.33 (22.42)	0.11 (14.10)	5.89 (28.46)

Key: *T0 *baseline, *T2 *week 12, *T3 *week 24, *M *mean, *SD *standard deviation, *n *number of participants who provided data at a given time point. T2–T0, T3–T2, T3–T0 represent change scores; change variables refer to participants who provided data at both time points. EORTCQLQ-30 scores range between 0 and100, with higher scores representing better well-being; Higher HADS scores indicate more distress, a score of ≥ 8 on either scale denotes anxiety or depression whilst scores above 11 are deemed abnormal; Fatigue scores can be categorised into mild fatigue (1–3), moderate fatigue (4–6), and severe fatigue (7–10); PA enjoyment scores range between 18 and 126 with higher scores representing greater PA enjoyment; The Self-efficacy for walking scale has a maximum score of 100

**Table 6:** MRS outcomes at 4 time points and associated change scores

	T0 (*n* = 28)	T1 (*n* = 24)	T2 (*n* = 19)	T3 (*n* = 18)	*T1-T0* (*n* = 24)	*T2-T1* (*n* = 17)	*T3-T2* (*n* = 18)	*T3-T0* (*n* = 18)
	M (SD)	M (SD)	M (SD)	M (SD)	M (SD)	M (SD)	M (SD)	M (SD)
*MRS*								
Quality of life	12.25 (8.35)	14.09 (7.79)	11.89 (7.80)	11.95 (8.62)	2.95 (5.31)	0.00 (5.27)	−0.83 (4.52)	−0.67 (8.51)
Psychological symptoms	4.67 (3.42)	5.74 (3.26)	4.56 (4.31)	4.95 (4.42)	1.62 (3.42)	−0.88 (2.55)	0.39 (2.20)	0.90 (4.14)
Urological symptoms	2.96 (2.76)	3.30 (2.85)	2.84 (2.24)	2.63 (2.79)	0.90 (2.10)	−0.33 (2.09)	−0.21 (2.18)	−0.26 (2.70)
Somatic symptoms	4.62 (3.42)	5.04 (3.97)	4.47 (3.36)	4.37 (2.91)	0.43 (3.16)	0.39 (3.22)	−0.11(2.88)	−0.05 (3.00)

### Mechanisms of impact

Concordant agreement between quantitative and qualitative data suggested that there was an impact of self-monitoring strategies and increased awareness of participant’s own behaviour, of their capabilities and of the PA guidelines on perceived self-efficacy for walking and PA enjoyment. It was also evident that despite in-person social interaction being impacted by the COVID-19 pandemic, online encouragement from other participants was found to be encouraging. Recognising the benefits of PA was a facilitator to programme participation as those who took time the time to reflect on the knock-on benefits found that this increased the value that they placed on PA:

I mean I'm not over the depression, but I feel like my days used to be really bad, where I would sit and cry. Now, I feel like I haven’t had a real bad day for…. I can't even remember …you still have your days when you think I can’t be bothered but I still move, even if it's just around the house trying to hit the 250 an hour (P11).

Unexpected mechanisms of impact were the development of habitual PA behaviours, with the integration of rituals (e.g. walking instead of driving; taking the stairs instead of the lift) into daily routines. Goal setting was broken down into a small change approach, making goals more achievable and which were spread throughout the week. PA time was perceived as a type of ‘self-care’ and a way for participants to prioritise their own health, separate from their caring responsibility roles.

When I do go out for these walks by myself, I love it so much. It might sound selfish to some people but it’s taking time for yourself because obviously if you’re not in the right state of mind, if you’re not looking after yourself then everyone is going to suffer around you (P3).

Barriers to behaviour change mainly related to factors that hindered social interaction between participants, in particular, the virtual platform and poor mental health. Barriers to walking included safety of the environment to walk alone: “By the time you've got the kids to bed and you’re both home together…. It's like seven o'clock and then I don't feel safe enough to walk, so it's hard” (P7). Other barriers were physical issues, such as hip pain and fatigue.

The COVID-19 pandemic created an unprecedented and ambiguous context for the evaluation of ACCEPTANCE. In particular, restrictions on travel and socialisation, and resulting poor mental health interfered with group walking and consequentially may have impacted the evaluation outcomes (e.g. QOL).

## Discussion

This is the first study to report the feasibility and acceptability of delivering and evaluating a PA programme (ACCEPTANCE) in CC survivors. The recruitment and retention rates for this study indicate that a future randomised controlled trial of ACCEPTANCE would be feasible, although as shown, self-reported PA levels may not be an appropriate eligibility criterion since achieving the national PA recommendations accounted for 50% of ineligibility. Alternative PA levels could be considered. One of the advantages of ACCEPTANCE is its individualised programme with 2-weekly health coaching sessions enabling goal setting to be tailored to participants’ individual circumstances. Evaluation of similar coaching sessions within behaviour change interventions has shown them to be a motivating factor for maintaining behaviour change [51, 52]. Previous research has demonstrated the feasibility of e-health technologies in combination with health coaching [53] and the current study builds on this to support its maintenance over a 12-week duration. Given that the diary component requires modification, it may also be viable to integrate such reflective and self-monitoring mechanisms into an e-health technology application to reduce the burden of an additional component completion. Emotional support received from fellow survivors is highly valuable [54]; however, peer support was not seen to be optimised in the intervention due to the virtual nature of many aspects and due to group walks requiring peer-led organisation. Given that in-person peer support was preferred by participants, focus on enabling participation in group walking via structured researcher-led organisation should be prioritised.

Results indicated that ACCEPTANCE has the potential to increase participants’ PA. The volume of PA increases of 1 mg (5–6 min of brisk walking) per day is meaningful [55], indicating that increases seen could equate to 20 min of brisk walking per day. Additionally, the increases in MVPA of 11.45 min per day from baseline to follow-up are considerably higher when compared to a change of + 6.1 min per day in a population of breast cancer survivors measured using hip-worn accelerometry [56]. The process evaluation found that such changes were attributable to expected mechanisms of self-monitoring and graded goal setting, along with unexpected mechanisms of integrating a small change approach [57] to goal setting and habit formation [58] to facilitate PA participation, which have been integrated into a refined logic model (Supplementary file S3.3).

Self-reported QOL scores were seen to decline throughout the study when measured using the QLQ-C30 global QOL scale with the follow-up score of 57.89 being comparable to patients undergoing treatment for CC [59, 60]. It must be noted that the study was conducted during the COVID-19 pandemic and participants qualitatively reported that their mental and social health was negatively impacted by restrictions on socialisation and travel. This suggests that declining QOL scores may be predominantly reflecting declines in social facets of QOL. This is further supported by improvements in anxiety and depression symptomology measured using the HADS from baseline to follow-up and such evidence was also reiterated via qualitative evaluation findings. However, baseline scores for both anxiety and depression were within the clinical range, suggesting that psychological and emotional well-being are a significant concern for this population, which is supported by findings from previous investigations [8, 9].

There are a number of conflicting results from the current study, such as a decline in menopause-related QOL and fatigue along with large improvements in insomnia symptoms, yet a decline in sleep duration. The variability in these results, including the decline in QOL scores may be attributable to a number of factors (e.g. natural fluctuations, COVID-19 restrictions and questionnaire use). Thus, further investigation which moves beyond that of feasibility testing, includes a control group and has the ability to account for treatment-related and sociodemographic factors is needed to ascertain any impact of the intervention alongside identifying potential confounders. Given that QOL is identified in the intervention development logic models, the implications of its measurement and conceptualisation should be considered carefully given its decline in the current trial. For example, the potential use of more frequent and rapidly administered ecological momentary assessment tools ought to be considered as a viable avenue to better gauge the nuanced well-being of participants in a definitive trial [61], helping to avoid inaccuracies which naturally arise from aggregated, past experiences [62]. Challenges related to reflecting on well-being were also highlighted by participants in relation to the diary completion, further emphasizing the need for a well-being measure which takes into account daily and weekly fluctuations.

This study’s results suggest there is interest in a behaviour change programme which can target CC survivors’ needs after treatment and that there are potential psychological and physical benefits of the ACCEPTANCE programme which can be further optimised subject to intervention refinement. This is in line with previous research findings that positive changes in health behaviours are associated with significant improvements in QOL in CC survivors [63]. Going forward, given that the majority of participants were of White British ethnicity (92.9%) which does not reflect the ethnic diversity seen in the UK CC population [1], strategies to engage and support CC survivors from ethnic minority groups should be prioritised. Such strategies could foster the inclusion of ethnic minority patients in a PPI, community or healthcare professional groups to understand specific barriers and facilitators to participation, alongside the utilisation resources which consider the communication needs (e.g. language, trust and relatability) of non-English speakers and recruitment strategies which target spaces frequented by ethnic minority groups [64, 65]. Whilst ACCEPTANCE may have the potential to increase PA levels and provide emotional support, greater and more structured targeting of peer support is recommended to potentially impact overall QOL (e.g. social health). A future randomised trial is needed to ascertain the effectiveness of the ACCEPTANCE intervention.

### Limitations

This feasibility trial was subject to some methodological limitations. Despite the programme evaluation benefitting from measures taken at multiple time points, there was noticeable attrition at the end of the programme (week 12). Change scores in outcomes represent differences between participants who completed measures at each time point, and thus comparison of outcomes is limited by less representation at week 12 to determine optimal programme length and to aid comparison of outcomes. In terms of feasibility evaluation, *Fitbit* compliance reported in this study refers to those who completed the evaluation questionnaire at week 12. An objective measure of *Fitbit* compliance throughout the programme would have provided greater insight into its acceptability. With regards to the process evaluation, despite 77% of participants taking part in an interview, only one of these participants had withdrawn, so it is likely that there are further barriers to participation that were not captured by these accounts.

## Conclusion

Only minor modifications in the study methods have been indicated before ACCEPTANCE can be adapted to a definitive randomised control trial. Recruitment and retention rates were deemed to be feasible, however, recruitment strategies and eligibility criteria require modification to increase the scope of the trial to offer the programme to those who may be excluded due to their PA levels and to those who are from ethnic minority groups. The study showed potential to increase physical activity, through hypothesised mechanisms of self-monitoring behaviour and problem solving, and unexpected mechanisms such as habit formation.

## Supplementary Information

Supplementary Material 1. CONSORT checklist of information to include when reporting a pilot trial*.

Supplementary Material 2: Table S2.1. Qualitative interview questions. Table S2.2. Compliance and completeness with evaluation measures at each time point. Figure S2.3. The logic model of the Problem.* NOTE*: PA= physical activity. Figure S2.4. The logic model of change. *NOTE: *PA= physical activity. Figure S2.5. Refined ACCEPTANCE logic model following process evaluation. *NOTE:* Content with a strike through has been removed; content in red font has been added.

## Data Availability

Data is unavailable due to ethical restrictions.
