# Supplementary material for: The feasibility and acceptability of delivering and evaluating a physical activity intervention (ACCEPTANCE) for cervical cancer survivors
Source: Pilot Feasibility Stud. 2025 Apr 5;11:42. doi: 10.1186/s40814-025-01622-8 (PMC11971866; doi:10.1186/s40814-025-01622-8)
Supplement: Supplementary file 2 — Supplementary Material 2: Table S2.1. Qualitative interview questions. Table S2.2. Compliance and completeness with evaluation measures at each time point. Figure S2.3. The logic model of the Problem. NOTE: PA= physical activity. Figure S2.4. The logic model of change. NOTE: PA= physical activity. Figure S2.5. Refined ACCEPTANCE logic model following process evaluation. NOTE: Content with a strike through has been removed; content in red font has been added. [file 40814_2025_1622_MOESM2_ESM.docx]

**Supplementary file S2- Tables**

| **Topic area** | **Questions** |
| --- | --- |
| Perceptions of the intervention | - How did you feel about the programme? - Did you enjoy taking part? / What did you enjoy the most?   - Prompts *(e.g. What was your experience of the programme launch?)* - Were there any aspects of the programme that you found hard to engage with?   - Prompts (e.g. *Why do you think you felt this way?)* - Do you think the programme could have been improved in any way?   - Prompts (e.g. *How do you do you think this change would affect your experience of the intervention?)* |
| Benefits of participation | - How do you feel now, having done the programme? - Would you recommend the intervention to a friend?   - Prompts (e.g. W*hich aspects in particular would you recommend?)* |
| Experience of group walking (where relevant/ if not previously mentioned) | - Why did/ didn’t you engage with the walking group? - How did you feel about walking in a group compared to by yourself?   - Prompts (e.g. *if you could go back, do you think you would engage more or less with the walking group?*) |
| Reasons for withdrawal (where relevant) | - Would you mind telling me about why you decided to stop doing the intervention? - Was there anything that could have helped you to keep going with it? |
| Maintenance of components | - Do you still use anything from the programme?   - Prompts (e.g. Why do you feel this aspect is beneficial/ Was It easy to keep using this after the programme?) |
| Maintenance of PA | - Have you noticed any differences in your PA since finishing the programme?   - Prompts (e.g. *How do you feel about that*?) |

**Table S2.1 Qualitative interview questions**

**Table S2.2 Compliance and completeness with evaluation measures at each time point**

|  | **Baseline (n=28)** | **Week-6 (n=26)** | **Week-12 (n=19)** | **Week-24 (n=18)** |
| --- | --- | --- | --- | --- |
| *Accelerometry* |  |  |  |  |
| No. Returned | 100% | 100% | 91.3% | 91.3% |
| Compliance | 100% | 92.3% | 87.5% | 78.3% |
| *Questionnaire measures* |  |  |  |  |
| Completeness |  |  |  |  |
| MRS | 89.2% | 73.1% | 100% | 100% |
| EORTCQLQ-C30 | 92.8% |  | 100% | 100% |
| FSI | 92.8% |  | 94.7% | 100% |
| HADS | 92.8% |  | 94.7% | 100% |
| SEW | 92.8% |  | 100% | 100% |
| PACES | 89.2% |  | 100% | 94.4% |

*KEY: EORTCQLQ-C30=* European Organisation for Research and Treatment of Cancer Quality of Life scale; *MRS=* Menopause Rating Scale*; FSI=* Fatigue Symptom Inventory*; HADS=* Hospital Anxiety and Depression scale; *SEW*= Self-Efficacy for walking scale; *PACES= Physical Activity Enjoyment Scale.*

| **Phase 4** |  | **Phase 3** |  | **Phase 2** |  |  |
| --- | --- | --- | --- | --- | --- | --- |
| Determinants |  | Behavioural factors |  | Health problems |  |  |
| - Low body confidence - Lack of knowledge regarding what physical activity they are capable of doing post treatment - Lack of knowledge of how to manage physical symptoms - Lack of knowledge regarding self-monitoring PA - Past experiences of PA |  | - Low PA levels - Low communication with practitioner regarding outcomes of treatment - Fear of recurrence - Unequipped to manage treatment after-effects |  | - Poor physical function - Bladder Dysfunction - Pelvic floor pain - Depression - Anxiety - Lymphedema - Poor sexual function |  | **Low quality of life in short and long term** |
|  |  |  |  |  |  |  |
| Environmental factors |  | Environmental factors |  |  |  |  |
| - Knowledge and support of partner and family members - Established interventions integrated into patient aftercare - Communication of treatment outcomes from medical professionals |  | - Lack of PA opportunities - Lack of specific interventions for cervical cancer patients - PA providers have a lack of knowledge regarding negative outcomes of cervical cancer treatment - Lack of knowledge among others causing a lack of relatedness |  |  |  |  |

**Figure S2.3**. The logic model of the Problem. *NOTE*: PA= physical activity

| **AT RISK GROUP** | | | | | | | | |
| --- | --- | --- | --- | --- | --- | --- | --- | --- |
| **Personal determinants and change objectives** |  | **Performance Objectives:** |  | **Behavioural Outcomes:** |  | **Health:** |  | **Quality of life improvement** |
| - Self- efficacy - Knowledge - Intrinsic motivation - Heath related benefits - Self- regulation - Implementation Intentions |  | - Decide to walk; Know / list benefits of walking - Plan when and where to walk; organise transport to the session; Identify comfortable clothes for walking - Monitor daily step count; know what counts as MVPA - Know what the PA guidelines are; Decide to achieve the guidelines. - Self- assess physical capabilities regularly; set specific, daily exercise goals |  | - Increased weekly step count - Increased PA goal setting - Time spent walking with similar others - Increased knowledge regarding PA benefits |  | - Improvements in psychological well- being, clinical anxiety and depression - Increased cardiovascular fitness levels - Improvements in physical health related to side effects of treatment/ self- management of side effects |  | - Decreased number of follow up appointments - burden on national health service |
|  | | | | | | | | |
| **Personal determinants and change objectives** |  | **Performance Objectives:** |  | **Environmental outcomes:** |  |  |  |  |
| - Self- efficacy - Knowledge - Implementation intentions |  | *Practitioners to:*   - Identify relevant and appropriate exercise information, express confidence in benefits of PA - Plan when to discuss PA; Listen for any barriers to PA - Know intervention related information |  | *Practitioners will:*   - Encourage PA participation - Know PA benefits |  |  |  |  |
| **ENVIRONMENT** | | | | | | | | |

| **AT RISK GROUP** | | | | | | | | |
| --- | --- | --- | --- | --- | --- | --- | --- | --- |
| **Personal determinants and change objectives** |  | **Performance Objectives:** |  | **Behavioural Outcomes:** |  | **Health:** |  | **Quality of life improvement** |
| - Self- efficacy - Knowledge - Intrinsic motivation - Heath related benefits - Self- regulation - Implementation Intentions |  | - Decide to walk; Know / list benefits of walking - Plan when and where to walk; organise transport to the session; Identify comfortable clothes for walking - Monitor daily step count; know what counts as MVPA - Know what the PA guidelines are; Decide to achieve the guidelines. - Self- assess physical capabilities regularly; set specific, daily exercise goals |  | - Increased weekly step count - Increased PA goal setting - Time spent walking with similar others - Increased knowledge regarding PA benefits |  | - Improvements in psychological well- being, clinical anxiety and depression - Increased cardiovascular fitness levels - Improvements in physical health related to side effects of treatment/ self- management of side effects |  | - Decreased number of follow up appointments - burden on national health service |
|  | | | | | | | | |
| **Personal determinants and change objectives** |  | **Performance Objectives:** |  | **Environmental outcomes:** |  |  |  |  |
| - Self- efficacy - Knowledge - Implementation intentions |  | *Practitioners to:*   - Identify relevant and appropriate exercise information, express confidence in benefits of PA - Plan when to discuss PA; Listen for any barriers to PA - Know intervention related information |  | *Practitioners will:*   - Encourage PA participation - Know PA benefits |  |  |  |  |
| **ENVIRONMENT** | | | | | | | | |

| **AT RISK GROUP** | | | | | | | | |
| --- | --- | --- | --- | --- | --- | --- | --- | --- |
| **Personal determinants and change objectives** |  | **Performance Objectives:** |  | **Behavioural Outcomes:** |  | **Health:** |  | **Quality of life improvement** |
| - Self- efficacy - Knowledge - Intrinsic motivation - Heath related benefits - Self- regulation - Implementation Intentions |  | - Decide to walk; Know / list benefits of walking - Plan when and where to walk; organise transport to the session; Identify comfortable clothes for walking - Monitor daily step count; know what counts as MVPA - Know what the PA guidelines are; Decide to achieve the guidelines. - Self- assess physical capabilities regularly; set specific, daily exercise goals |  | - Increased weekly step count - Increased PA goal setting - Time spent walking with similar others - Increased knowledge regarding PA benefits |  | - Improvements in psychological well- being, clinical anxiety and depression - Increased cardiovascular fitness levels - Improvements in physical health related to side effects of treatment/ self- management of side effects |  | - Decreased number of follow up appointments - burden on national health service |
|  | | | | | | | | |
| **Personal determinants and change objectives** |  | **Performance Objectives:** |  | **Environmental outcomes:** |  |  |  |  |
| - Self- efficacy - Knowledge - Implementation intentions |  | *Practitioners to:*   - Identify relevant and appropriate exercise information, express confidence in benefits of PA - Plan when to discuss PA; Listen for any barriers to PA - Know intervention related information |  | *Practitioners will:*   - Encourage PA participation - Know PA benefits |  |  |  |  |
| **ENVIRONMENT** | | | | | | | | |

**Figure S2.4**. The logic model of change. *NOTE:* PA= physical activity

| **UNDERPINNING MODEL AND THEORIES** |  | **OUTPUT ACTIVITIES** |  | **SHORT-TERM OUTCOMES** |  | **LONG-TERM OUTCOMES** |
| --- | --- | --- | --- | --- | --- | --- |
|  |  |  |  |  |  |  |
| The intervention is grounded in several behaviour change theories and has been developed using the Intervention Mapping protocol |  | Participant enrolment |  | Compliance with intervention |  | A larger definitive pilot trial |
|  |  |  |  |  |  |  |
|  |  | Education session on PA and goal setting session |  | Completion of evaluation measures |  |  |
| **Individual and group:** Grounded in Social Cognitive Theory (SCT) and theories of Self-Regulation. Intervention functions: Education, implementation, self-monitoring of behaviour, Self-efficacy for PA |  |  |  |  |  |  |
|  |  | 12-weeks of a self-monitoring activity monitor; daily prompts to increase PA and review goals; diary completion |  | Recruitment, retention and attrition |  |  |
|  |  |  |  |  |  |  |
| **Environmental:** Grounded in SCT and theories of Self- Regulation, ~~the health belief model~~ and habit theory. Intervention functions: Self-monitoring, relatedness, observational learning; habit formation |  | Fortnightly health coaching and problem solving, goal setting; small change approach and goal review |  | The feasibility of the inclusion criteria |  |  |
|  |  |  |  |  |  |  |
|  |  | Peer support via group walking with similar others |  | Trends in PA levels |  |  |

**Figure S2.5.** Refined ACCEPTANCE logic model following process evaluation. *NOTE:* Content with a strike through has been removed; content in red font has been added
